# Nutritional implications for ultra-endurance walking and running events

**DOI:** 10.1186/s13728-016-0054-0

**Published:** 2016-11-21

**Authors:** Eric Williamson

**Affiliations:** Department of Exercise Science, University of Toronto, 55 Harbord Street, Toronto, ON M5S 2W6 Canada

**Keywords:** Ultra-endurance, Energy, Nutrition, Performance, Training

## Abstract

This paper examines the various nutritional challenges which athletes encounter in preparing for and participating in ultra-endurance walking and running events. Special attention is paid to energy level, performance, and recovery within the context of athletes’ intake of carbohydrate, protein, fat, and various vitamins and minerals. It outlines, by way of a review of literature, those factors which promote optimal performance for the ultra-endurance athlete and provides recommendations from multiple researchers concerned with the nutrition and performance of ultra-endurance athletes. Despite the availability of some research about the subject, there is a paucity of longitudinal material which examines athletes by nature and type of ultra-endurance event, gender, age, race, and unique physiological characteristics. Optimal nutrition results in a decreased risk of energy depletion, better performance, and quicker full-recovery.

## Background

As a crucial aspect of the life of athletes, and a basic element of physical fitness, endurance is significantly impacted by not only physiological characteristics but very importantly, the body’s capacity to effectively utilize nutrients to sustain performance, particularly during ultra-endurance events. Defined as events lasting at least 6 h [1], ultra-endurance events place extreme and unique physiological demands on athletes. Some events span several days, including those that have no scheduled breaks [2]. The diversity in location in which these events are sometimes performed presents athletes with unique challenges including extreme temperatures, increase in altitudes, rapid energy depletion, and the need to consume nutrients during the event. Proper training is important to prepare for such extraordinary physical feats, but nutrition is paramount as these events would not be possible without adequate fuel availability. Nutrition, hydration, and recovery are among the most important considerations for athletes, which require advanced planning.

It has been identified that a comprehensive source providing succinct guidelines and recommendation to both protect the health of these athletes and promote performance is not available. Numerous case reports and field studies [3–24] show that few ultra-endurance runners and walkers meet recommendations that have been established throughout the literature. In an observational study of 42 amateur runners in a Swiss mountain marathon, researchers discovered that the intake of most participants were significantly below the requisite nutritional recommendations [7]. They further asserted that 90% of ultramarathon runners agreed that nutrition has an important influence on overall performance. This being said, adequate food and fluid intake is related to a successful finish of an ultra-endurance race [7, 24] and an important key to attaining this adequacy seems to be an appropriate nutrition strategy during the race [25]. These findings are possible indicators that the difficulty which athletes experience in meeting standard recommendations could be attributed to various factors. Among these are lack of or poor nutrition education, norms of ultra-endurance sports, the development of physical symptoms including injury, gastrointestinal disturbances, suppression of appetite, logistic challenges with implications for both food preparation in terms of time and available resources/facilities to do so and, by extension, total food intake particularly in those periods of increased needs [15, 26, 27]. Additionally, dehydration and fluid overload [14, 26–29] appear to be areas with which have challenges. The myriad of stressors, such as extreme environmental conditions, intense physical exertion, limited sleep, and rationing of food, which ultra-endurance athletes encounter [14, 26–29], highlights the importance of prior planning where individualized nutrition strategy is concerned. It is clearly demonstrated throughout the literature that there is a need for appropriate education of ultra-endurance athletes, coaches, medical staff and race organizers, based on environmental conditions and course topography. Overarching goals should be aimed at minimizing the energy gap between intake and expenditure, attaining adequate dietary intakes of micronutrients and avoiding over or under hydration. This review will comprehensively discuss recommendations to address these issues.

## Review

### Energy needs of the ultramarathon athlete

As can be seen in Table 1, ultra-endurance events are highly diverse, but available literature suggest that they result in an energy deficit. Ultra-endurance athletes typically train for 1–6 h per day and many have multiple training sessions per day [30]. It is not uncommon to train for longer than 6 h at a time as some events require more than 24 h of continuous activity [30]. Therefore, as shown in Table 1, quantities of energy intake well above those of the average person are required to fuel the activity for both training sessions and events. With performance as a primary goal, athletes should strive to achieve an energy intake that matches the energy output of their activity, basal metabolic rate (BMR), thermic effect of food, and other activities of their daily life. It is important that ultra-endurance athletes consider these variables both during activity and outside the context of activity as failure to restore energy between training sessions can delay recovery and be detrimental to performance. Extreme energy deficits have been found to be a common feature among athletes who engage in continuous and multi-stage ultramarathon events. This is subsequently associated with poor recovery from exercise and sustained fatigue [14, 15]. Both inadvertent symptoms such as gastrointestinal challenges and injury, including those that are dermatologically related, increase the risk of insufficient food and fluid intake with and without the addition of environmental challenges [31, 32]. In competition, field research suggests that ultra-endurance athletes finish their races with an energy intake between 36 and 54% of energy expenditure [18, 34]. With this in consideration, Ramos-Campo et al. [33] have found that the magnitude of the energy deficit is correlated with performance, which suggests that reducing this energy deficit may be an advantage.

**Table 1:** A comparison of ultra-endurance walking and running events

Year published	Name	Subject(s)	Location	Time length	Exercise mode	Distance (km)	Energy intake	Energy expenditure	Total energy deficit	Climate	Terrain/altitude	Nutrition/fluid stations	Self-procured nutrition	Reference
1977	N/A	1 male	N/A	20 h	Running and walking	160	9600 kcal	10,720 kcal	−1120 kcal	Temperature range: 12–20 °C Wind speeds range: 15–17 km/h	N/A	Fluid stations	None	[16]
1993	N/A	9 female	N/A	7 days	Running	N/A	14,270 kcal	20,940 kcal	−6670 kcal	N/A	N/A	N/A	Food: ad libitum Liquids: ad libitum	[23]
1994	N/A	1 male	Sydney to Melbourne, Australia	199 h	Running	1005	5972.57 kcal/day	N/A	N/A	Temperature range: 8–25 °C	Accumulated altitude: 900 m Ascent: 1000 m Descent: 100 m	Food and beverage provided every 15–20 mins. Fluids: carbohydrate supplemented beverage Food: Potato, rice, pasta, and bread	N/A	[21]
2000	Australian run	1 male	Australia	217 days (data compiled over 2 weeks of this time)	Running	14,500	N/A	6321 kcal	Negative	N/A	N/A	None	Food: ad libitum Fluids: ad libitum	[12]
2002	N/A	26 (21 male, 5 female)	New York, USA	26.2 ± 3.6 h	Running	160	7050 kcal	14,340 kcal	7290 kcal	Temperature range: 21–38 °C	N/A	37 food stations	None	[8]
2004	Marathon Des Sables	1 male	Sahara Desert	6 days	Running	229	17,572 kcal	33,776.75 kcal	–16,204.745 kcal	Temperature range: 5 °C(night)–50 °C(day)	N/A	None	Food: ad libitum (dehydrated meals) Fluids: ad libitum (carbohydrate supplemented drinks)	[195]
2005	Tour des Dents du Midi	42 (39 male, 3 female)	Switzerland	7 h 3 min	Running and walking	44	219–2405 kcal	N/A	−1889 to −2470 kcal	Temperature range: 18–30 °C Humidity range: 34–61% at the lowest altitude, and 57–92% at the highest altitude Wind speeds range: 1–21 km/h	Total ascent:2890 m Ascent and descent range: 860–2494 m	15 support stations Fluids:water, sweet tea Food: Banana and orange slices, dried fruit mix, cereal bars and grape sugar cubes	Food: ad libitum Fluids: ad libitum	[7]
2010	100 km Biel	11 female	Biel, Switzerland	12.7 h ± 91 min	Running	100	570 ± 230 kcal	6310 ± 1340 kcal	−5750 ± 1170 kcal	Temperature range: 8–15 °C	N/A	17 aid stations Fluids: isotonic sports drinks,tea, soup, caffeinated drinks and water Food: bananas, oranges, energy bars and bread	Fluids: ad libitum	[17]
2011	N/A	1 male	Atcama Desert, Chile	23 days	Walking	593	40,733 kcal	110,791 kcal	–70,058 kcal	Described as: temperate climate	Average altitude: 3103 ± 704 m	None	Food: freeze-dried foods, snacks Beverages: coffee	[13]
2011	100 km Biel	27 male	Biel, Switzerland	11.5 h ± 119 min	Running	100	760 ± 300 kcal	7420 ± 1660 kcal	–6660 ± 1650 kcal	Temperature range: 8–18 °C	N/A	17 aid stations Fluids: isotonic sports drinks, tea, soup, caffeinated drinks and water Food: bananas, oranges, energy bars and bread	Fluids: ad libitum	[20]
2013	MSUM	74 (46 male, 28 female)	Al Andalus Ultimate Trail, Spain	5 days	Running	225	16,740 kcal	19,155–24,995 kcal	–2415 to –8225 kcal	Described as: hot ambient environment	N/A	Aid stations situated 10 km apart Food: fruit (oranges and watermelon) Fluids: plain water, electrolyte supplementation.	Fluids: ad libitum	[15]
2013	MDUER	1 male	North Scotland to Moroccan Sahara desert	78 days	Running	4254	5541.2 ± 764.3 kcal/day	N/A	N/A	Described as: extreme weather conditions (maximum range: 2.8–45.0 °C)	Altitude of ascent and descent ranged between 0 and 2400 m above sea level, with 7 days at altitude ^3^1500 m	Food and liquid provided daily	N/A	[9]
2014	Glenmore24 Trail Race	25 (19 male, 6 female)	Cairngorms National Park, UK	24 h	Running and walking	122–208	4776.9 ± 2627.3 kcal	13,136.5 ± 2627.3 kcal	–8359.6 kcal	Temperature range: 0–20 °C in 2011 and 3–19 °C in 2012 Humidity range: 54–82%	Average altitude: 342 m (SD 303 m)	Plain water and electrolyte supplementation every 3 km	Food: ad libitum Fluids: ad libitum	[14]
2014	N/A	6 unspecified	Sierra de Gredos, Spain	14 h 6 min	Running	54	5124.6 ± 531.2 kcal	9856.6 ± 859.8 kcal	–4732 kcal	Temperature range: 8–26 °C Temperature average: 14.9 ± 8.7 °C	Maximum altitude : 2484 m Minimum altitude : 1149 m	None	Food: energy bars Fluids: water from various natural sources	[10]
2015	South Pole Race	13 (12 male, 1 female)	Antarctic	22.5 days	Running	800	Faster finishers: 5332 ± 469 kcal/day Slower finishers: 3048 ± 1140 kcal/day	N/A	N/A	Temperature average: −24.0 °C Humidity: 59.3% Wind speed: 6.6 ms	Altitude range: 2000–2615 m	Snack bags provided (contents: macadamias, chocolate bars, cheese, candy, biltong, muesli, freeze-dried meals, noodles, soup, hot chocolate, coffee, tea, milk)	Food: ad libitum Fluids: ad libitum	[11]
2016	N/A	11 unspecified	Castles of Cartagena, Spain	6 h 44 min ± 28 min	Running	54	1493.1 ± 491.5 kcal	5197.1 ± 488.8 kcal	–3704 kcal	N/A	Accumulated altitude: 5391 m;	None	Food: energy bars, glucose tablets and fruit Fluids: Water and energy drinks	[33]

Table 1 A comparison of ultra-endurance walking and running events.

As in standard marathon runners, attaining an intake that is as close as possible to energy output should be a noteworthy ambition [35]. Both general and environment/activity-specific implications and strategies on how to do this will be discussed in the following sections. However, it should be recognized that other non-nutritional strategies to reduce the risk of inadequate energy intake, such as those to reduce gastrointestinal symptoms and injuries, play a role in achieving this. Common GI challenges that hinder intake include nausea, abdominal cramping, bloating, diarrhea, vomiting, flatulence, and belching [26, 36]. These issues are more common as intensity and/or duration increase. Common injuries that hinder intake depend largely on the environment and climate and include blisters, subungual haematomas, chafings, abrasions, and plantar fasciitis [26, 37]. Climate and environmental-specific injuries include blisters and sunburns in hot temperatures, [26] and frostnip and frostbite in cold temperatures [37].

#### Carbohydrate

Given that the majority of an ultra-endurance athlete’s training is spent engaged in lengthy durations of aerobic activity, many of these athletes are well adapted to utilizing lipids via oxidative phosphorylation [35]. However, the energy demands of their specific activity will vary, predominantly depending on the duration, intensity and type of exercise being engaged in [38]. Intensity, duration, and food intake will largely determine how much fuel is being sourced from carbohydrates (CHO), protein, and fat. Although all three are being used as sources of energy at any given time, the intensity and duration are primary factors which determine the extent to which one is used over another. When the athlete is exercising at the standard marathon pace that requires 80–90% of maximal oxygen consumption (VO_2_ max) or above, carbohydrate will be his or her primary fuel source and could provide up to 96% of the energy being expended [35]. However, at lower intensities in which sufficient oxygen can be achieved, such as walking, much more fuel could be provided from fat [39]. Therefore, the fraction of macronutrient utilization distribution is of considerable dependence on individual and exercise differences as well as carbohydrate availability, with lower availability forcing the body to depend more highly on fat and protein.

Based on the preceding discussion, as well as the observation that elite marathon running is nearly 100% CHO-dependent [40], awareness of CHO intake is important during training and events, especially those for which completion in minimal time is an objective. In fact, many studies have demonstrated that increases in the hourly rate of CHO and overall energy intake are correlated with faster race times in ultra-endurance events [8, 18, 41]. This suggests that athletes should strive to maximize availability of CHO for their working muscles and reinforce the need for adequate energy to maintain performance.

Glycogen provides a reserve of CHO for the body and low glycogen availability appears to be a stimulus for feelings of fatigue [42]. To maximize fuel storage as glycogen for events, a high carbohydrate diet is generally suggested between training sessions and events [43]. Current recommendations regarding specific recommendations for carbohydrate ingestion have recently been reviewed by Burke and Hawley [44]. Specifically, 8–12 g of CHO/kg body weight/day is recommended, with a more precise amount dependent on the athlete’s training intensity and duration [45]. This being said, the need for high carbohydrate intakes both before and during the event is dependent on whether carbohydrate fuel sources are depleted or limiting for the demands. Increases in intensity, duration, demand of terrain [45], experience level of the athlete [40], and altitude [46, 47], all, increase carbohydrate needs. It is not a concern of athletes’ about consuming too much as almost all ultra-endurance walking and running events result in a deficit (as shown in Table 1) and narrowing the gap between energy intake and expenditure correlates positively with performance, rather it is a question of whether to pack carbohydrates or fat as the fuel source if they are carrying their own food. Fat provides more energy per gram and if the above variables are towards the lower end and less carbohydrate is needed, packing foods higher in fat will make the athletes carry load lighter and could allow them to narrow the energy gap further. This will be discussed in further detail in the section discussing dietary fat.

Current practices suggest that carbohydrate intakes in the diets of ultra-distance athletes range from 5 to 7 g/kg/day in regular diets during training to 7–10 g/kg/day during the 3–4 days prior to competition [48]. A study by Mahon et al. [49] on mountain ultramarathon runners found that despite over 65% of athletes reporting that they intended to increase their CHO intake in the week prior to the event, no participants came close to their CHO-loading recommendations of 10–12 g/kg/d in the 48 h leading up to the event. This demonstrates that although a high carbohydrate intake is well known to benefit long duration endurance performance, athletes often fail to reach daily CHO targets needed to maximize glycogen storage due to the difficulty in practical application. As carbohydrate intakes both prior to and during ultra-endurance events with demanding characteristics of those discussed above are positively correlated with performance, athletes should strive to consume as close to this recommendation as possible if needed. Possible means of doing so is through frequent consumption of carbohydrate dense foods that are low in highly satiating nutrients, mainly being water, protein, and fiber [50], and high on the glycemic index. Examples include white rice, pretzels, breakfast cereals, bagels, and granola bars.

In addition, to restore glycogen stores between exercise sessions, a carbohydrate intake of 1.0–1.5 g/kg at 2 h intervals for the first 6 h and beginning within the first 30 min following exercise appears to be an effective strategy for recovery [51]. Consumption of carbohydrates during performance has also been shown to be beneficial to best conserve muscle and hepatic glycogen storage and to maintain blood glucose concentration. A carbohydrate intake as high as 90 g/h for the extensive duration of activities being discussed is suggested to maintain performance [43]. Again, however, this appears to have practical difficulties. Mahon et al. found that the average intake of the ultramarathon mountain runners was just 28 g/h. Another study on a 100 km ultra endurance running race found that mean intake was only 43 g/h. Again, narrowing the gap between energy intake and energy expenditure results in improvements in performance and athletes should strive to increase this g/h intake. Some ways in which athletes may be able to achieve this is through fluids, gels, and even whole foods, depending on the athlete’s preferences and gastrointestinal tolerance. Experimenting with different forms of carbohydrate in fluid replacement beverages such as glucose, maltose, fructose polymers, and branched chain starches with high glycemic indices at a concentration of 6–12% are recommended to provide carbohydrate late in exercise as muscle and liver glycogen stores become depleted and the risk of hypoglycemia is increased [52, 105]. These carbohydrates can also be provided in gel or bar form as it was recently demonstrated that carbohydrates in a beverage are oxidized at similar rates to carbohydrates from a gel [53] and from a bar [54]. Further ways to increase intake during events through management of gastrointestinal symptoms (GIS) will be discussed in the section on gastrointestinal intolerances.

#### Fat

Dietary fat is essential for optimal health and should not be overlooked by those engaging in ultra-endurance events. For those consuming a medium to high carbohydrate diet, a fat consumption similar to that recommended for the general population of 20–35% of energy intake is generally suggested to maintain performance and health [43]. Endurance training is known to enhance an athlete’s capacity for fat oxidation during exercise and fat oxidation provides the greatest relative contribution to energy expenditure during low to moderate intensities of exercise with a peak recently shown to occur at 64 ± 4% VO_2_ max [55]. Recent research has explored ways in which this can be further up-regulated to enhance exercise capacity and sports performance by reducing the reliance on the muscles’ limited glycogen stores and need to consume carbohydrate during prolonged events. Strategies employed to attain this include consuming a very low carbohydrate (<50 g/day) high fat (>70% of energy consumption) diet for either scheduled periods or permanently [56]. After 2–3 weeks on this diet, the body is able to adapt to using fat at greater contributions, sparing more carbohydrate [57].

With a reduced reliance on carbohydrates as a fuel source as well as the elimination of the need to consume carbohydrates during activity, many potential advantages are presented. The athlete would no longer be required to carry sources of CHO with him or her, worry about attaining enough CHO or risk GIS from eating during activity. However, this strategy also comes at a cost. This reliance on fat limits the intensity of exercise that can be performed and severely restricts the capacity to do anaerobic work [57, 58]. This is due to the decreased availability of CHO for glycolysis, the body’s fastest energy producing mechanism for intense work.

In a study on mountain ultramarathon runners, Mahon et al. [49] found that those consuming suboptimal amounts of CHO had higher levels of blood β-ketones post-event and that these post-blood β-ketone levels were negatively associated with performance. This further supports the need for CHO intake during prolonged events, given that ketones are an indicator of fat metabolism, particularly if an objective is to complete the event in minimal time. It is also important to note that in non-fat adapted athletes low CHO availability increases muscle protein breakdown [59] and if performed chronically can lead to a loss of skeletal muscle mass. However, naturally during multi-day events, exercise pacing tends to conform to submaximal levels of intensity, often below lactate threshold to preserve limited glycogen stores and optimize fat utilization and the Krebs cycle pathway for ATP resynthesis [60]. This being said, fat adaptation is worth experimenting with for those who consume far below the recommended intakes of energy and carbohydrates for their events, particularly for those who are prone to GIS. Bringing calorie intake closer to energy expenditure using fat also improves performance when compared to a larger caloric deficit without extra fat [61, 62]. Since fat is more calorically dense than protein and carbohydrate, athletes who must carry their own food should choose high fat food options if it allows them to reach closer to their caloric needs over carbohydrate. Therefore, this strategy may be most appropriate for those competing in ultra-events which have breaks and which athletes must carry their own food.

Although preloading with dietary fat, specifically medium chain triglycerides (MCT), has strong literature support to potentially improve performance based on its capacity to serve as a fuel source and spare muscle glycogen [63, 64], the majority of studies have found no glycogen preserving effect or improvement in shorter distance endurance performance [65–70]. In longer duration activities, the research is conflicting. A study by Van Zyl et al. [193] found that performance in cyclists who rode for greater than 2 h in a 40 km simulated time trial had greater performance with supplemented beverages containing CHO+MCT during the trial rather than either CHO or MCT alone. Contrary to this, Jeukendrup et al. [67] also studied long duration cycling activity (180 min) and found that the contribution to energy expenditure was small and did not provide any significant benefit to performance or carbohydrate preservation. The difference in the results of these two studies is likely due to the quantity of MCT ingested by the participants. Van Zyl et al. provided 86 g in total whereas Jeukendrup et al. provided 29 g in total. However, an intake of 86 g far exceeds the recommended maximum by many authors (30 g) who suggest intakes higher than this lead to gastrointestinal discomfort and diarrhea [71–73]. A later study by Jeukendrup et al. [74] attempted to test an intake of 85 g and found that it did indeed decrease performance due to provocation of GIS. At this time, the literature does not support the use of MCT supplementation in ultra-endurance activity.

#### Protein

Protein is a critical nutrient requiring considerable attention by the athlete to ensure proper recovery from exercise and to promote optimal adaptation between training sessions. The protein needs of athletes engaging in prolonged activity are greater than those required for the general population because of the need to repair damaged muscles and synthesize new muscle proteins. It further serves as an energy substrate during activity [75]. The repair and generation of body proteins greatly contribute to athletes’ sought after adaptations to induced challenges and consequent improvements in performance.

Bodily protein stores have been shown to provide up to 10% of the total energy used during endurance exercises [76]. The fraction of contribution is influenced by many factors including intensity, duration and, as previously discussed, the level of glycogen/glucose availability in the body [76, 77]. When it comes to increased metabolic efficiency with training, a certain degree of metabolic efficiency does occur to mitigate amino acid oxidation with training [95], however, the rate of oxidation still increases over 2 h of endurance activity resulting in a several fold increase compared to resting conditions regardless of training level [96, 97]. Due to both the use of amino acids as a fuel source as well as muscle damage associated with exercise, skeletal muscle mass seems to decrease in ultra-endurance running events without breaks, as has been shown in a few case reports of ultra-endurance athletes [3, 78]. In contrast, in ultra-endurance events where there are breaks, skeletal muscle mass tends to remain stable [79–81]. When muscle loss occurs from walking or running, with the exception of the thigh, it has been shown to occur in all muscle groups with the greatest losses occurring in the lower leg or calf region [3, 82, 83]. The eccentric contractions involved in running cause the greater portion of body mass lost as muscle mass comparatively [82] to more concentric-based ultra-endurance activities such as cycling [84]. One way in which athletes may reduce the amount of endogenous protein lost, and by extension, promote recovery, is by ensuring adequate glycogen stores going into exercise and by consuming adequate energy during prolonged activity [35]. The following recommendations can also help ensure athletes are recovering lost muscle and preventing loss of skeletal muscle mass during training and events.

While a vast body of research supports a “hypertrophy-centric” view following resistance exercise, recent research highlights a critical role for dietary protein in supporting recovery from endurance exercise. Although the pre-eminent adaptations in resistance exercise compared to endurance exercise may be different, the requirements for amount, type, and timing are similar [75]. Protein remodeling, which is primarily determined by changes in muscle protein synthesis, is an important aspect of the acute recovery process after exercise that ultimately underpins the adaptations (e.g., greater muscle power, aerobic capacity) that accrue with endurance training [75]. Numerous studies have reported increases in mixed muscle protein synthesis following a single bout [85, 86] of exercise, and both short-term (i.e., 4 weeks) [87] and chronic (i.e., 4 months) [88] endurance training. Such increases in mixed muscle protein synthesis likely reflect enhanced remodeling of muscle proteins that may include mitochondrial-related proteins/enzymes, angiogenic proteins (e.g., endothelial and smooth muscle cells within capillaries), and myofibrillar proteins.

The current recommended intake of protein is 1.2–2.0 g/kg for a general athletic population [45]. Given the extraordinary caloric needs to fuel these unique tasks, it is likely that these athletes are meeting and possibly exceeding this recommendation if they are meeting their energy requirements [76]. In addition to daily protein needs, other factors are also important for optimizing performance adaptations, including timing and partitioning of intake. To maximize protein synthesis, and thus muscle remodeling and recovery [89], it is suggested that endurance athletes consume a minimum of 20 g of protein at 3–4 h intervals to maximize muscle protein synthesis [75, 90]. The amount required for ultra-endurance athletes and those who exercise longer than 2 h is presently unclear. However, it is likely that their needs would be even higher given the increase in total oxidation of amino acids during exercise as well as the possibility of splanchnic organ tissue damage due to the shunting of blood away from the digestive system during activity [91]. The rate of muscle breakdown is accelerated when muscle protein oxidation exceeds synthesis, which usually occurs in proportion to intensity and duration of the sporting activity [92–94].

Currently, ultra-endurance runners consume an approximate average of 12% of energy as protein during racing [98]. It has been posited that supplemental protein or amino acids on top of this intake during an ultra-run may improve performance through provision of amino acids for use as a fuel source and to attenuate muscle damage [99]. Despite the use of supplementary amino acids having been shown to improve performance and decrease muscle soreness in cyclists, a study on ultramarathon runners showed no benefits. Knechtle et al. [100] supplemented 14 subjects with 52.5 g of amino acids immediately before and during a 100 km run and compared them against a placebo group. Contrary to their hypothesis, there were no improvements in performance or effects on parameters related to skeletal muscle damage in the supplemented group. Unfortunately, measures of skeletal muscle damage were only taken immediately after the race. More research is needed to determine if the intake of amino acids during the race would lead to lower values of these markers in the following hours and days of recovery. Therefore, at the present time, evidence would suggest no additional benefit from consuming supplementary amino acids or protein during ultra-endurance running events.

In comparison to resistance exercisers, the immediacy of dietary protein intake after exercise is critical for optimal recovery [101, 102]. The consumption of a snack or meal with a minimum of 20 g of protein within 30–60 min post exercise is suggested to optimally stimulate muscle protein synthesis and attenuate any existing breakdown that is ongoing from the bout of prolonged exercise [75].

#### Hydration

As little as a 2% reduction in body mass due to dehydration has been said to result in performance decrements as well as hemorheology, metabolic dysregulation, heat intolerance, and cardiovascular strain [103]. However, weight changes before and after an ultra-distance event do not provide an accurate indication of hydration status and weight loss greater than 2% does not necessarily have serious adverse consequences on performance [104]. Hoffman et al. [104] found that in addition to hydration status being unrelated to changes in weight, runners in a 161 km ultramarathon had a mean weight loss of approximately 3% and that many of the top performers had a weight loss of beyond 2% for much of the race. In other activities such as shorter duration endurance events, hydration needs for an event can be approximated during training through methods such as taking body weight before and after training at a duration, intensity, and environment that mimics that of a competition [105]. However, because reductions in body mass can be attributed to substantial breakdown of body tissues such as adipose and muscle [11] and increases in weight can result from reduced diuresis as well as decreases in intracellular osmolytes including glycogen, proteins, and triglycerides, this would be an ineffective strategy for ultra-endurance athletes. The reduced diuresis is induced by activation of vasopressin secretion and the angiotensin–renin–aldosterone mechanism during exercise and the decreases in intracellular osmolytes causes a shift of water to the extracellular compartment during very prolonged exercise [106]. With the complexity of hydration during these events, hyper-hydration has become increasingly common and is the most reported medical complication to occur during ultra-distance triathlons [107]. This is crucial as this can lead to the life-threatening case of hyponatremia by altering the blood serum to sodium ratio [108]. In fact, this shift appears to be a primary result of fluid overload and is unrelated to sodium losses [109]. To prevent over or underhydration, current available research suggests that the most suitable strategy to maintain hydration is to *‘drink to thirst’* [15, 27, 104, 109–112].

**Fig. 1:**
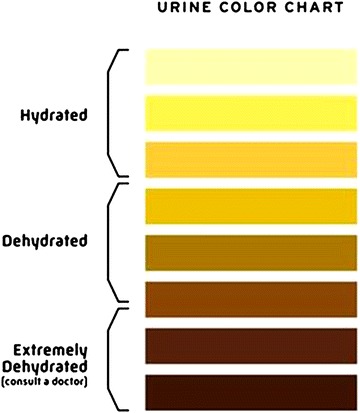
Urine color as an indication of hydration status (reproduced with permission from [196])

Urine color (see Fig. 1) can also be used to guide hydration in ultra-endurance running. However, it should be noted that urine concentration (i.e., color and osmolarity) rises substantially throughout the race and increasingly becomes less reliable with duration [15]. Costa et al. found that it is in fact less reliable than relying on thirst as an indicator of hydration status [15]. It is important to note here that substrate metabolism is also altered as a result of dehydration during exercise resulting in greater reliance on carbohydrate as a fuel source [113]. Although the fatigue associated with dehydration is mainly a result of hyperthermia it also results in lower FFA uptake and higher muscle glycogen utilization [114]. Therefore, not only is maintaining hydration important for sustaining an optimal body temperature, preventing immediate fatigue, but it is also important to spare glycogen, potentially preventing or delaying later onset of fatigue.

Because sweat also contains sodium one might argue that sodium supplementation may be of importance during ultra-endurance walking and running events. Published data has shown that as high as 90–96% of ultra-endurance runners use sodium supplements [27, 29, 104]. Although past recommendations suggest a sodium intake of 1.7–2.9 g/L of fluid consumed to allow for fluid retention, more recent data have shown no benefit to hydration [19, 27–29, 104, 115] or blood serum sodium levels [19, 28, 29, 115] by consuming supplemental sodium during these races. This is likely due to the adaptations that increase sodium bioavailability and prevent losses (e.g. sweat, urine, and feces) which take place in response to periods of sodium deprivation or restriction [115–117]. In fact, sodium supplements taken in excess can result in inadequate weight loss and even unnecessary weight gain [118]. This ultimately results in fluid overload and decrements to performance as discussed above. It is therefore recommended that to best maintain hydration, athletes drink to thirst without using sodium supplementation beyond that taken in food and fluids, even when exercising in high ambient temperatures [104].

Other recommendations for maintaining euhydration during the event pertain to both the use of carbohydrate supplemented beverages and fluid intake before the event. As mentioned in the carbohydrate section, a concentration of 6–12% of carbohydrate is recommended for those that consume carbohydrate-supplemented beverages to achieve rapid absorption, reduce the risk of cramping, and provide energy [52, 105]. At 2–4 h prior to exercise, to achieve hydration balance going into the event, it is recommended to consume 5–10 mL/kg body weight from water or carbohydrate-supplemented beverage. This will allow enough time for excretion of any excess as urine before the event allowing for a balanced bodily fluid level going into the activity [45].

#### Vitamins and minerals

Vitamin and mineral considerations are crucial when participating in and training for ultra-endurance activities. When it comes to athletic performance, these micronutrients are particularly important for energy production, hemoglobin synthesis, maintenance of bone health, adequate immune function, and protection of the body against oxidative damage. They also assist in important physiological processes related to synthesis, recovery, and adaptation to exercise. Because of this, exercise may increase the turnover and loss of these nutrients resulting in greater dietary intakes being required. Some vitamins and minerals that athletes need to pay particular attention to are calcium, vitamins D, C, E, and the B vitamins, iron, zinc, magnesium, as well as, beta carotene and selenium for their antioxidant properties.

Calcium and vitamin D play important roles in growth, maintenance, and repair of bone tissue as well as regulation of nerve conduction, and development and homeostasis in skeletal muscle. A deficiency in both or either calcium and vitamin D increases the risk of low bone-mineral density and stress fractures [119]. Calcium can be obtained from food; however, vitamin D is mainly synthesized through sunlight. Serum Vitamin D levels should be tested regularly, especially in athletes who do not receive adequate sunlight daily, such as those who live at northern latitudes (>35th parallel) or who primarily train indoors throughout the year [120]. In those with suboptimal levels (stated in Table 2), supplementation may be necessary. Current vitamin D supplement recommendations suggest 1000–2000 IU per day for athletes [121].

**Table 2:** Optimal serum levels for ultra-endurance runners/walkers

Micronutrient	Serum marker	Optimal serum level
Vitamin C	L-Ascorbic acid	40-60 μM
Calcium	Calcium	4.5–5.5 mEq/L
Vitamin D	25-hydroxyvitamin D	75–100 nmol/L
Vitamin E	Alpha Tocopherol	5.5–17 µg/mL
Folate	Plasma folate	2.7–20 μg/L
Vitamin B12	Holotranscobalamin	35–156 pmol/L
Iron	Ferritin	>50 ng/mL
Magnesium	Magnesium	1.5–3.0 mEq/L
Zinc	Zinc	84–159 µg/dL

B vitamins play a role in energy production and the building and repair of muscle tissue. There is some data suggesting that to obtain optimal health and performance, highly active athletes may need to double the current recommended amounts of these B vitamins though it is likely that these needs are being met with increased energy intakes [122]. Of particular consideration, however, are vitamin B12 and folate. A deficiency in either of these nutrients results in anemia which can greatly reduce time to fatigue and therefore endurance performance [123]. Because vitamin B12 is obtained through animal products, such as meat and dairy, athletes such as vegetarians or vegans may need to consume supplements with this vitamin.

Iron deficiency will also result in anemia, reducing the ability of red blood cells to transport oxygen. A deficiency in iron is common among those engaged in prolonged activity due to up-regulation of the hormone hepcidin. The increase in this hormone is observed hours after exercise and reduces the gut’s ability to absorb dietary iron [124]. Because of this, ultra-endurance athletes should pay particular attention to their iron consumption and obtain regular blood tests to check their ferritin status. Iron absorption can be improved by consuming heme iron found in meat products with non-heme iron found in plant products and vitamin C with sources of iron [125, 126]. Athletes should aim for blood ferritin levels of >50 μg/L for optimal performance and iron supplements may be considered under the discretion of a health care provider if this level is not being met through dietary sources alone [127, 128].

Zinc plays a role in muscle repair, energy metabolism, and immune status. A deficiency in zinc can result in disrupted thyroid hormone levels, affecting metabolic rate and performance [129]. It can also reduce cardiorespiratory function, muscle strength, and endurance [123]. Athletes are at high risk of inadequate zinc levels [130] and should therefore strive to achieve adequate zinc intake through zinc-rich foods. They should be cautioned if using zinc supplements that they do not exceed the tolerable Upper Intake Level (UL) of zinc (40 mg/day) [131], which can lead to decreases in high-density lipoprotein cholesterol and nutrient imbalances by interfering with the bioavailability of other minerals such as iron and copper [123]. Zinc-rich foods include shellfish, green leafy vegetables, and seeds. If supplementation is required, athletes should receive guidance from their health care provider.

Magnesium supports the proper functioning of the nervous and musculoskeletal systems [132]. Deficiency can cause multiple symptoms resulting in decreased performance as it is linked to many pathological conditions of the cardiovascular, skeletal, and nervous systems [133]. Ultra-endurance athletes are at increased risk of this deficiency due to increased urinary and sweat losses induced by magnesium redistribution within the body during prolonged intense activity [134]. In addition, the dietary reference intake of 310–420 mg/day is likely suboptimal for most athletes [135]. Ultra-endurance athletes should have their blood levels of magnesium tested regularly and self-monitor for common symptoms of hypomagnesaemia such as muscle cramps. Supplementation with magnesium is recommended if necessary and dosage should be determined under the discretion of their healthcare provider to avoid toxicity.

#### Antioxidants

Exercise can induce a release of free radicals or reactive oxygen species which have the ability to modify lipids, proteins, carbohydrates, and nucleic acids in the body [136]. These modifications are collectively known as oxidative damage or oxidative stress and have been linked to negative health outcomes such as insulin resistance, atherosclerosis, cardiac dysfunction, and injury [137]. Antioxidant vitamins and minerals, such as vitamins C and E, beta carotene, and selenium can be used to mitigate these effects. These nutrients act in different ways to either remove oxidative species or prevent their reactions from happening [138]. However, because oxidative species also have some beneficial effects on the body, their function is not to completely eliminate these processes, but to keep them at homeostatic, and thus optimal, levels. Therefore, there is a threshold to which antioxidants can provide benefits for performance, health, and recovery. Research on ultra-endurance athletes has demonstrated that their need to prevent oxidative damage is higher given their extraordinary exercise volume [136].

Although more research is needed to examine the effects of these antioxidant supplements during and immediately prior to an event, current evidence suggests little to no benefit [139, 140]. A study on runners ingesting vitamin supplements (*N* = 9) and mineral supplements (*N* = 12) showed that the supplementation did not result in faster race times compared to the athletes without supplemental intake of vitamins and minerals [141]. It is important to note that although ultra-endurance athletes may benefit from ample intakes of antioxidant vitamins and minerals that exceed the current recommendations for the general population, they should be cautioned not to consume these nutrients at levels above the ULs. High doses above the UL can also result in pro-oxidative effects, causing risks of decreased performance, recovery and health [142].

Other antioxidants which have recently been investigated for their effects on endurance performance include polyphenols with the most popularly researched being quercetin, catechins, and resveratrol. These polyphenols are organic chemical compounds mainly found in plants that have strong antioxidant properties [143]. They have also been shown to have anti-inflammatory, cardioprotection, and anti-carcinogenic properties in clinical populations [144]. However, few studies have investigated the effects of these polyphenols on performance, particularly in an ultra-endurance population.

Catechins are commonly found in plants such as green tea and cacao. Some human studies have shown positive effects for endurance including V02 max [145], fat oxidation, and insulin sensitivity [146] in an untrained population; however, studies on trained subjects are yet to show benefits [147–149]. It is unlikely that supplemental catechins would be beneficial to ultra-endurance performance.

Resveratrol is present in concentrated quantities in grapes. It’s strong antioxidant properties have shown to be beneficial against degenerative and cardiovascular diseases from atherosclerosis, hypertension, ischemia/reperfusion, heart failure, diabetes, obesity, aging, and neurodegenerative diseases [150]. With one exception, studies to date have only been performed on rodents, and the effects on performance range from extremely beneficial to extremely detrimental [151–157]. Taken together, these studies would suggest that resveratrol benefits trained rodents and is potentially harmful in untrained rodents. The only human study was performed in untrained elderly participants and the effect demonstrated that supplementation was also potentially harmful through blunting of cardiovascular training adaptations to endurance exercise [151]. Further research is needed before supplemental resveratrol should be taken by ultra-endurance athletes.

Quercetin is found in foods such as red onion, dill, apples and capers and has been studied more extensively than other polyphenols. It provides many health benefits in humans [158] and has shown to encourage mitochondrial growth in rodents [159]. Although quercetin supplementation shows potential endurance performance benefits in cell culture and in vivo animal studies [160, 161], research on its use as a supplement in humans are less clear. Some studies have reported increased endurance exercise capacity and performance in humans following supplementation with quercetin [162–164]; however, many have failed to find benefits [165–171]. Of the 2 studies [172, 173] on ultra-endurance trained subjects, both have shown no significant benefit. Nieman et al. [172] examined the effect of quercetin supplementation on inflammation after three consecutive days of cycling and following an ultra-endurance run. No improvements in performance or attenuation of markers of muscle damage, inflammation, increases in plasma cytokines, and alterations in muscle cytokine mRNA expression were found [172]. Quindry et al. [173] supplemented half of their 63 ultra-endurance running trained subjects with quercetin combined with niacin and vitamin C for 3 weeks leading up to and during a 160 km ultramarathon. The supplement did not fortify plasma antioxidant levels against ultramarathon-induced oxidative stress in blood plasma or improve performance. This being said, a 2011 meta analysis by Kressler et al. [194] encompassing the above research concluded that quercetin supplementation can improve human endurance exercise capacity in a small but significant magnitude (~3%). Based on data showing favorable outcomes for supplemental quercetin [162–164], a daily dosage of 1000 mg could have small potential benefits and is unlikely to be detrimental for ultra-endurance trained populations.

Where micronutrients in general are concerned, there are currently no Recommended Dietary Allowance (RDA)’s in place specifically for athletes. However, the amounts needed in excess of those recommended for the general population are likely dependent on multiple factors including individual variability, training intensity, and training duration. To determine if ultra-endurance athletes are consuming adequate amounts of vitamins and minerals, they should obtain regular blood tests to ensure blood levels are being maintained at levels that are not only acceptable for general health but are optimal for performance (see Table 2). This may be particularly important during times when their training or nutrition changes. It is important to emphasize that regular adequate intake of vitamins and minerals is required for optimal performance and that consuming extra vitamins and minerals through supplementation immediately before or during an ultra-endurance event has not shown to provide any performance, health or recovery benefits [141, 174].

### Gastrointestinal intolerances

During ultra-endurance activities and corresponding training exercises, food and fluid must be consumed while being active to minimize the energy deficit. Because of this, it is no surprise that GIS are a common issue for these athletes [175]. Endeavoring to prevent GIS is important as it is one of the most common cited reasons for inadequate intake during events [176, 177] and is positively correlated with increasing duration [178]. Running in particular appears to result in more pronounced GIS than other activities [175] as well as a dehydrated state compared with a euhydrated state [179]. There also seems to be an individual predisposition for GI distress during exercise as Pfieffer et al. have determined a positive relationship between GIS during races and history of GI issues both associated with and away from exercise [175, 180]. Another common issue in ultra-endurance athletes is reduced appetite, which is closely related to GIS as both are subsequent results of splanchnic ischemia. Particularly at workloads above 70 % VO_2_ max, splanchnic blood flow is reduced to about 30–40% as blood shifts to working muscles and skin to dissipate heat [177].

If the event has no enforced breaks, whole foods may not be an option as they may be too difficult to chew and swallow and could result in GIS. In this case, intake from fluids is a viable option as not only does it provide the energy but also hydration. However, in cases where the prevention of hyper-hydration is important, products such as sports gels can also be supplemented to the racer’s diet. With gels, it has been shown that high doses of CHO (1.4 g/min) are well tolerated by most runners [180]. Against this background, it may be best to determine strategies, such as use of different types of nutritional sources and frequency of consumption to find which methods work best to maximize carbohydrate intake during an event without causing GI distress. One of the possible ways that this could be done is through coingestion of glucose and fructose as a carbohydrate source rather than one or the other. Research suggests that this can increase carbohydrate oxidation from an average of 1–1.26 g/min mainly due to increased bioavailability as the 2 different compounds use different transporters within the gut [181]. With the use of gels as a source of carbohydrates, Pfeiffer et al. [180] showed no overall difference in tolerance between glucose-based gels and combined glucose and fructose gels. However, some individuals showed more symptoms with one or the other gel. It should, therefore, be advised that individual athletes, especially those who experience GI problems frequently, test their tolerance during intense training sessions, ideally under conditions similar to those of the races they aim to compete in.

The intake of the nutrients fat, fiber, and protein, have all been linked to GIS during exercise [182]. To prevent this, food items low in these nutrients, such as bananas, biscuits, energy gels/bars, and sports drinks, are popular food and fluid choices for ultra-endurance events. However, as the duration of ultra-endurance races increases, these food and drink choices have become less tolerable and appealing [183, 184]. In terms of athletes’ tolerance, individual testing of food and drink intake during training conditions similar to the event they are training for are vital. No matter where the athlete is starting from, another potential strategy is “gut training”, which involves increasing the absorptive capacity of the gut through high carbohydrate dieting and progressively increasing the hourly carbohydrate intake during training [185]. Although the evidence of this is mainly anecdotal, intestinal carbohydrate transporters can indeed be up-regulated [186, 187] and gastric emptying rates can be enhanced with training [188].

GIS occur less frequently after adequate training or when relative exercise intensity is reduced [189, 190]. Although more research in this area is needed, experimentation with this strategy during training is likely to present little risk and athletes should dedicate at least some time to gut training. Endurance training itself appears to enhance gastric transit time [191], and higher energy intakes during training further enhance this rate [192]. Cox et al. [187] demonstrated that exogenous carbohydrate oxidation rates were higher after the high carbohydrate diet (6.5; 1.5 g/kg BW provided mainly as a carbohydrate supplement during training) for 28 days compared with a control diet (5 g/kg BW/day) in endurance trained cyclists. The higher rates were attributed to improved absorption, which provides evidence that the gut is indeed adaptable and that this could be used as a practical method to increase exogenous carbohydrate oxidation. Therefore, ultra-endurance runners should strive to gradually increase their intakes as tolerated during training to further approach suggested intakes (kcals/km) for events. This could lead to improvements in performance through greater fuel availability as discussed in preceding sections.

## Conclusion

There is a paucity of agreed-on and concrete nutrition best practices for ultraendurance runners and even less demarcating such by event type. From a macronutrients perspective, ultra-endurance athletes need to ensure adequate intake. Generally, carbohydrate, protein, and fat recommendations are 8–12 g of CHO/kg body weight/day, ≥20 g at 3–4 h intervals and 20–35% of energy intake, respectively, and athletes should strive to minimize the gap between energy intake and energy expenditure to optimize performance. However, the practicality of such recommendations needs to be considered on an individual basis and the importance of rehearsal of an individualized nutrition strategy prior to competition cannot be overemphasized. Because micronutrients are crucial and may sometimes be overlooked, special attention needs to be placed on each both in terms of interaction with the body’s internal physiology, other ingested foods and the nature and intensity of physical rigor the body endures. As far as is necessary, and in keeping with advice from healthcare providers, ultra-endurance athletes may use supplements to support training and events performance and aid in recovery. While some recommendations presented are prescriptive in nature based on the findings of various studies, ultra-endurance athletes are encouraged to apply them within the context of their particular training regiment, body mass composition, and corresponding physiological needs. All the literature reviewed indicate that ultra-endurance athletes must take great care in attending to their nutritional needs to maintain good health, promote optimal performance, and reduce the likelihood of injuries. Proper nutrition will result in decreased energy depletion, better performance, and accelerated recovery. With the growing international appeal of ultra-endurance events, significant research is needed to promote the health and wellbeing of athletes. More longitudinal studies are needed to ascertain the precise nutritional and environmental conditions under which athletes perform most optimally based on age, gender, type of event, body type, and other physiological factors.

## Abbreviations

BMR: basal metabolic rate
CHO: carbohydrates
UL: upper intake level
RDA: recommended dietary allowance
GIS: gastrointestinal symptoms
ATP: adensosine triphospate
